# Risk factors associated with amyotrophic lateral sclerosis based on the observational study: a systematic review and meta-analysis

**DOI:** 10.3389/fnins.2023.1196722

**Published:** 2023-05-22

**Authors:** Qiaochu Zhu, Jing Zhou, Yijie Zhang, Hai Huang, Jie Han, Biwei Cao, Dandan Xu, Yan Zhao, Gang Chen

**Affiliations:** ^1^Department of Acupuncture and Orthopedics, Hubei University of Chinese Medicine, Wuhan, China; ^2^Department of Tuina and Rehabilitation Medicine, Hubei Provincial Hospital of Traditional Chinese Medicine, Wuhan, China; ^3^Department of Tuina and Rehabilitation Medicine, Affiliated Hospital of Hubei University of Chinese Medicine, Wuhan, China; ^4^Department of Tuina and Rehabilitation Medicine, Hubei Institute of Traditional Chinese Medicine, Wuhan, China; ^5^Department of First Clinical Medical College, Hubei University of Chinese Medicine, Wuhan, China; ^6^School of Sports Medicine, Wuhan Sports University, Wuhan, China

**Keywords:** amyotrophic lateral sclerosis, risk factors, observational study, meta-analysis, systematic review

## Abstract

**Objective:**

Amyotrophic lateral sclerosis (ALS) is a fatal neurodegenerative disorder affecting the upper and lower motor neurons. Though the pathogenesis of ALS is still unclear, exploring the associations between risk factors and ALS can provide reliable evidence to find the pathogenesis. This meta-analysis aims to synthesize all related risk factors of ALS to understand this disease comprehensively.

**Methods:**

We searched the following databases: PubMed, EMBASE, Cochrane library, Web of Science, and Scopus. Moreover, observational studies, including cohort studies, and case-control studies, were included in this meta-analysis.

**Results:**

A total of 36 eligible observational studies were included, and 10 of them were cohort studies and the rest were case-control studies. We found six factors exacerbated the progression of disease: head trauma (OR = 1.26, 95% CI = 1.13, 1.40), physical activity (OR = 1.06, 95% CI = 1.04, 1.09), electric shock (OR = 2.72, 95% CI = 1.62, 4.56), military service (OR = 1.34, 95% CI = 1.11, 1.61), pesticides (OR = 1.96, 95% CI = 1.7, 2.26), and lead exposure (OR = 2.31, 95% CI = 1.44, 3.71). Of note, type 2 diabetes mellitus was a protective factor for ALS. However, cerebrovascular disease (OR = 0.99, 95% CI = 0.75, 1.29), agriculture (OR = 1.22, 95% CI = 0.74, 1.99), industry (OR = 1.24, 95% CI = 0.81, 1.91), service (OR = 0.47, 95% CI = 0.19, 1.17), smoking (OR = 1.25, 95% CI = 0.5, 3.09), chemicals (OR = 2.45, 95% CI = 0.89, 6.77), and heavy metal (OR = 1.5, 95% CI = 0.47, 4.84) were not risk factors for ALS based on meta-analyses.

**Conclusions:**

Head trauma, physical activity, electric shock, military service, pesticides, and lead were risk factors for ALS onset and progression. But DM was a protective factor. This finding provides a better understanding of ALS risk factors with strong evidence for clinicians to rationalize clinical intervention strategies.

**INPLSY registration number:**

https://inplasy.com/inplasy-2022-9-0118/, INPLASY202290118.

## 1. Introduction

Amyotrophic lateral sclerosis (ALS) is a fatal motor neuron disease (MND) affecting the upper and lower motor neurons in the spinal bulb, cerebral cortex, and spinal cord (van Es et al., 2017). Muscle atrophy and dysfunctions are the most common symptoms of this neurodegenerative disease (Loeffler et al., 2016; Pender et al., 2020). There are four subtypes of MND based on clinical symptoms, including amyotrophic lateral sclerosis (ALS), progressive muscular atrophy (PMA), progressive bulbar palsy (PBP), and primary lateral sclerosis (PLS) (Aiello et al., 2022). With the progression of the disease, PMA, PBP, and PLS will eventually lead to ALS, the most common type of MND (Walters et al., 2019). The epidemiology of ALS indicates that the incidence rate is estimated to be ~2.6 cases per 100 000 individuals annually (Xu et al., 2020). About 80% of patients who suffer from the disease die within 5 years due to respiratory muscle failure, but only 10% of cases can survive for more than 10 years from diagnosis (Talbott et al., 2016; Longinetti and Fang, 2019). ALS may not only result in high readmission rates and dysfunctions such as limb paralysis, decreased respiratory function, and muscle atrophy, but it also has a significant negative impact on the psychological and financial burden of patients and relatives (Conroy et al., 2021).

Nowadays, the etiology and pathogenesis of ALS remain unclear. Therefore, no effective cure has yet been found for this condition. USA Food and Drug Administration approved the only drug, riluzole, a glutamatergic neurotransmission inhibitor that can slightly benefit survival (Jaiswal, 2019). Riluzole only prolongs the life span for 3–5 months, costing $1,000 per month (Thakore et al., 2020). Consequently, most patients are treated in a palliative capacity and live with the situation (Salzmann et al., 2022). Baumann et al. (2019) pointed out that the emergence of ALS probably involves multifactorial interactions of complex mechanisms. Due to the debilitating nature of the disease, identifying risk factors in ALS is essential, which is of great significance for a comprehensive understanding of the epidemiological characteristics of ALS and scientific prevention and treatment. There is currently some evidence for specific risk factors for ALS, including elderly age (Chen et al., 2015), smoking (Calvo et al., 2016), physical activity (Harwood et al., 2016), military service (Seals et al., 2016a), diabetes mellitus (DM) (Cui et al., 2021), and alcohol consumption (Huisman et al., 2015). However, some studies have reported conflicting results (Huisman et al., 2015; Kioumourtzoglou et al., 2015). Given these controversial results and the small sample sizes of several studies, we aimed to explore risk factors in ALS by collecting data from published research in electronic databases in the past decade to obtain a reference value based on a large sample with high reliability.

## 2. Materials and methods

This meta-analysis strictly adhered to the Preferred Reporting Items for Systematic Reviews and Meta-Analyses (PRISMA) guidelines (McInnes et al., 2018). There was no need for ethical approval because this was a systematic review. The protocol has been registered in the INPLSAY platform with the number INPLASY202290118.

### 2.1. Search strategy

We searched the following electronic databases PubMed, EMBASE, Web of Science, Cochrane library, and Scopus to identify relevant studies from January 2012 to June 2022, using a combination of MeSH terms and keywords: “Amyotrophic Lateral Sclerosis,” “risk factors,” “case-control studies,” “cross-sectional studies,” and “Cohort Studies.” Meanwhile, we comprehensively searched observational clinical trials including cohort and case-control studies, which are ongoing via the WHO International Clinical Trials Registry Platform (WHO ICTRP) and ClinicalTrials.gov. We also screened the references of included studies in order to avoid missing eligible studies that have not been retrieved by the search strategy. Preprint servers (such as medRxiv and Research Square) were searched for unpublished data. The search strategy of PubMed is shown in Table 1. The rest of the databases comply in the same way.

**Table 1:** Search strategy of PubMed.

**Search number**	**Query**	**Results**
#1	“Amyotrophic Lateral Sclerosis”[Mesh]	21,990
#2	((((((((Sclerosis, Amyotrophic Lateral[Title/Abstract]) OR (Gehrig's Disease[Title/Abstract])) OR (Gehrig Disease[Title/Abstract])) OR (Charcot Disease[Title/Abstract])) OR (Motor Neuron Disease, Amyotrophic Lateral Sclerosis[Title/Abstract])) OR (Lou Gehrig's Disease[Title/Abstract])) OR (ALS - Amyotrophic Lateral Sclerosis[Title/Abstract])) OR (ALS Amyotrophic Lateral Sclerosis[Title/Abstract])) OR (Lou Gehrig Disease[Title/Abstract])	692
#3	#1 or #2	22,355
#4	(relative[Title/Abstract] AND risk^*^[Title/Abstract]) OR (relative risk[Text Word]) OR risks[Text Word] OR cohort studies[MeSH:noexp] OR (cohort[Title/Abstract] AND stud^*^[Title/Abstract])	1,102,056
#5	#3 and #4	1,309

### 2.2. Inclusion and exclusion criteria

Observational studies include cross-sectional studies, case-control studies, and cohort studies. For the part of cross-sectional studies and case-control studies, the inclusion criteria for the meta-analysis were as follows: (1) the original studies reported the relationship between specific risk factors and ALS, as well as odds ratio (OR) and 95% confidence interval (CI); (2) participants were diagnosed with ALS by professional medical institutions regardless of nation, age, sex, or race; (3) healthy dwellers or patients without neurodegenerative disease were assigned to the control group; (4) NOS assessment with higher than 7 scores. For the part of cohort studies, the original studies were required to provide adjusted relative risk (RR) or hazard ratio (HR) and 95% CI, and other criteria were the same as for the case-control studies.

The exclusion criteria were as follows: (1) ALS patients accompanied by other neurodegenerative diseases; (2) systematic review, randomized controlled trials, case reports, animal research, and conference papers; (3) repeated publications and data-missing studies; (4) studies with no risk factor reported, or studies reported risk factors but no OR, RR; (5) unreasonable statistical methods; (6) the NOS quality assessment below seven stars.

### 2.3. Study selection

All retrieved studies were managed with NoteExpress 3.0 software. Firstly, two independent reviewers excluded studies not meeting the inclusion criteria after reading the title and abstract. And then, we downloaded the rest of included studies and read the full text to check which studies followed the inclusion criteria. We reached a consensus by discussing any disagreements in the systematic review process with a third reviewer.

### 2.4. Data extraction and quality assessment

Two reviewers independently extracted the following items of included studies: title, published year, first author, type of study design, source of cases and controls, numbers of patients and controls, risk factor (s), OR, RR, HR, and CI. After extracting data, two investigators cross-checked the data. For the potential studies with missing data, we contacted the corresponding author for raw data via email. Any disagreement would be resolved by discussion.

Two independent reviewers assessed the quality of case-control and cohort studies by the Newcastle-Ottawa scale (NOS) (Stang, 2010). A case-control or cohort study in NOS involves three domains (Selection, Comparability, and Exposure) covering four, two, and three points. High-quality research gains more than seven stars. Only more than seven points were considered high-quality research. This systematic review mainly included high-quality studies (a NOS score higher than seven stars) to improve the reliability of evidence. Scoring details for all included studies were shown in Supplementary Table 1.

### 2.5. Statistical analysis

All risk factors for ALS have been extracted from original studies. If more than two observational studies reported a specific risk factor, we would perform meta-analyses based on this factor. The primary outcome is the ORs of ALS by possible risk factors in case-control studies and RRs/HRs of ALS risk factors in cohort studies. *I*^2^ statistics assess heterogeneity (Higgins et al., 2003). *I*^2^ > 75% is recognized as significant heterogeneity, 50% < *I*^2^≤75% is recognized as moderate heterogeneity, 25% < *I*^2^≤50% is recognized as low heterogeneity, and *I*^2^≤25% was recognized as homogeneity. We used the fixed effects model if heterogeneity was low or homogeneous. Otherwise, we choose the random effects model. Sensitivity analysis was performed to detect the source of heterogeneity. Every time we remove a potential heterogeneous study, we must explain the reason. STATA15.0 was operated in the statistical analyses mentioned above.

## 3. Results

### 3.1. Study selection and characteristics

**Figure 1:**
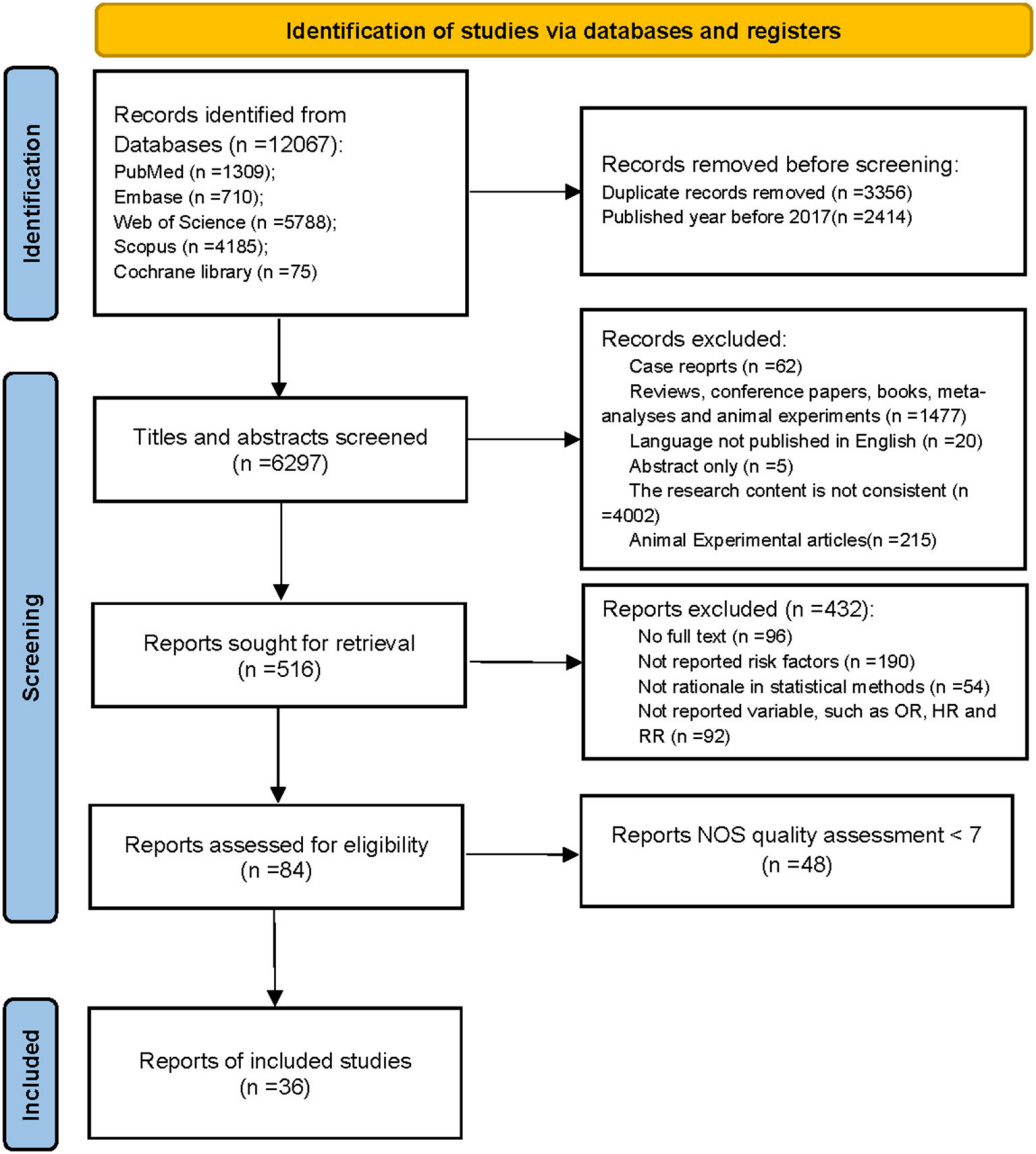
Flow diagram of selection process.

Database searches resulted in 12,067 studies, which were initially considered for this meta-analysis. A total of 5,770 studies were excluded due to duplication and year discrepancies. By reviewing the titles and abstracts, another 5,781 studies were excluded since case reports, non-English literature, letters, inconsistent content, or conferences did not meet the inclusion criteria. After reading the full texts, 432 studies were excluded because the contents were unavailable, risk factors were not reported, statistical methods were not reasonable, and ORs or RRs were not reported. Finally, the remaining 84 studies were scored by NOS, and 48 were below seven scores excluded. Thereinto, 36 studies, which were scored above seven scores, were finally included in the meta-analysis. The study selection process is shown in Figure 1. The characteristics of the included studies are shown in Table 2.

**Table 2:** Baseline of included studies.

**Author**	**References**	**Publication year**	**Study type**	**Cases**	**Controls**	**Source of controls**	**Risk factor**
Kamalesh2012	Das et al. (2012)	2012	Case-control	110	240	Non-neurodegenerative disorders people	④, ⑥, ⑦, ⑪, ⑭, ⑲, ㉘
Tracy2012	Peters et al. (2013)	2012	Cohort	3,888	19,632	Dweller	①
Elinor2013	Fondell et al. (2013)	2013	Cohort	42	40,046	Dweller	㉒
Yu2014	Yu et al. (2014)	2014	Case-control	66	66	Non-neurodegenerative disorders patients	⑤, ⑥, ⑨, ㉘
Meinie2014	Seelen et al. (2014)	2014	Case-control	722	2,268	Dweller	①, ②, ③
Feng2015	Lin et al. (2015)	2015	Case-control	729	14,580	Dweller	⑫
Ching2015	Tsai et al. (2015)	2015	Case-control	729	7,290	Dweller	⑯, ㉓
D.Mariosa2015	Mariosa et al. (2015)	2015	Case-control	224	1,437	Non-ALS patients	③
Marianthi2015	Kioumourtzoglou et al. (2016)	2015	Case-control	516	47,787	Dweller	②
Marianthi2015	Kioumourtzoglou et al. (2015)	2015	Case-control	55	9,239	Dweller	③
Mark2015	Huisman et al. (2015)	2015	Case-control	674	2,093	dweller	㉖
Angela2015	Malek et al. (2015)	2015	Case-control	66	66	Non-ALS patients	⑪
Yu2015	Sun et al. (2015)	2015	Cohort	615,492	614,835	Non-DM patients	③
Ryan2016	Seals et al. (2016b)	2016	Case-control	116	8,922	Dweller	⑧
Su2016	Su et al. (2016)	2016	Case-control	156	128	Healthy control	⑧, ⑨, ⑩, ⑬, ⑰
Peters2016	Peters et al. (2016)	2016	Case-control	163	229	Dweller	㉔
Harwood2016	Harwood et al. (2016)	2016	Case-control	317	715	Dweller	⑥
Tracy2016	Peters et al. (2017)	2016	Case-control	59	227	Dweller	④
Yvonne2016	Eaglehouse et al. (2016)	2016	Cohort	28	121	Non-DM patients	⑥
Ryan2016	Seals et al. (2016b)	2016	Case-control	3,650	365,000	Dweller	①
Anne2018	Visser et al. (2018)	2018	Case-control	1,557	2,922	Dweller	⑥
D'Ovidio2018	D'Ovidio et al. (2018)	2018	Cohort	76,279	651,698	Non-DM patients	③
Ola2019	Nakken et al. (2019)	2019	Cohort	2,968	1,465,282	Dweller	㉑
Tommaso2020	Filippini et al. (2020)	2020	Case-control	95	135	Dweller	④, ⑦, ⑩
Andrew2021	(Andrew A. S. et al., 2021)	2021	Case-control	188	376	Dweller	①, ⑦, ⑩
Andrew2021	(Andrew A. S. et al., 2021)	2021	Case-control	500	1,949	Healthy control	⑨
Yu2021	Yu et al. (2021)	2021	Case-control	1,636	4,024	Healthy control	⑮
Rosenbohm2021	Rosenbohm et al. (2021)	2021	Case-control	393	791	Dweller	④
Skajaa2021	Skajaa et al. (2021)	2021	Cohort	852	974,304	Dweller	⑳
Thompson2021	Thompson et al. (2022)	2021	Cohort	294	429,710	Dweller	㉗
Sun2021	Sun et al. (2021)	2021	Cohort	483,442	2,392,647	Healthy control	㉕
Beaudin2022	Beaudin et al. (2022)	2022	Case-control	403	378	Healthy control	①, ⑨
Mitsumoto2022	Mitsumoto et al. (2022)	2022	Case-control	95	106	Healthy control	⑨, ⑩
Andrew2022	Andrew et al. (2022)	2022	Case-control	553	762	Healthy control	⑩
He2022	He et al. (2022)	2022	Cohort	140	203	Healthy control	⑱, ㉙
TE2022	Wang et al. (2023)	2022	Cohort	36	99	Not exposed to lead	⑱

① head trauma; ② CVD, cerebrovascular disease; ③ DM, diabetes mellitus; ④ occupation; ⑤ smoking; ⑥ physical activity; ⑦ electricity shock; ⑧ military service; ⑨ pesticide; ⑩ lead; ⑪ chemical agent; ⑫ hypotensor; ⑬ educational status; ⑭ residential; ⑮ air pollution; ⑯ steroid; ⑰ service industry; ⑱ high metabolism; ⑲ water resources; ⑳ statin; ㉑ BMI, body mass index; ㉒ alopecia; ㉓ aspirin; ㉔ trace metals in blood; ㉕ intestinal biopsy; ㉖ nutrition; ㉗ lipid metabolism; ㉘ heavy metal; ㉙ FVC, forced vital capacity.

### 3.2. Meta-analyses of risk factors in ALS

#### 3.2.1. Head trauma

**Figure 2:**
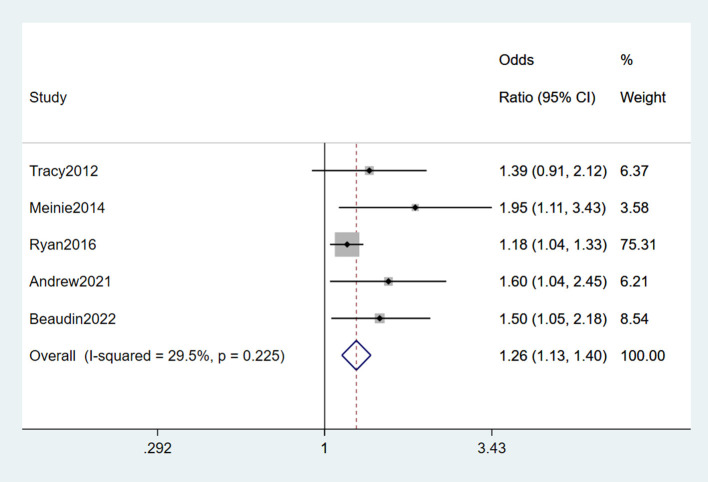
Forest plot of head trauma.

Among included studies, five (Peters et al., 2013; Seelen et al., 2014; Seals et al., 2016a; Andrew A. et al., 2021; Beaudin et al., 2022) reported head trauma as a risk factor for ALS, one (Peters et al., 2013) of which was a cohort study, and the rest were case-control studies. From the forest plot, slight heterogeneity was detected (*I*^2^ = 29.5%). Therefore, the fixed-effects model was performed for the meta-analysis of head trauma, and it was a risk factor for ALS (OR = 1.26, 95% CI = 1.13, 1.40, *P* = 0.225), which indicated that a 1.26 times higher risk of developing ALS with head trauma than without experiencing head trauma (Figure 2).

#### 3.2.2. Cerebrovascular disease

**Figure 3:**
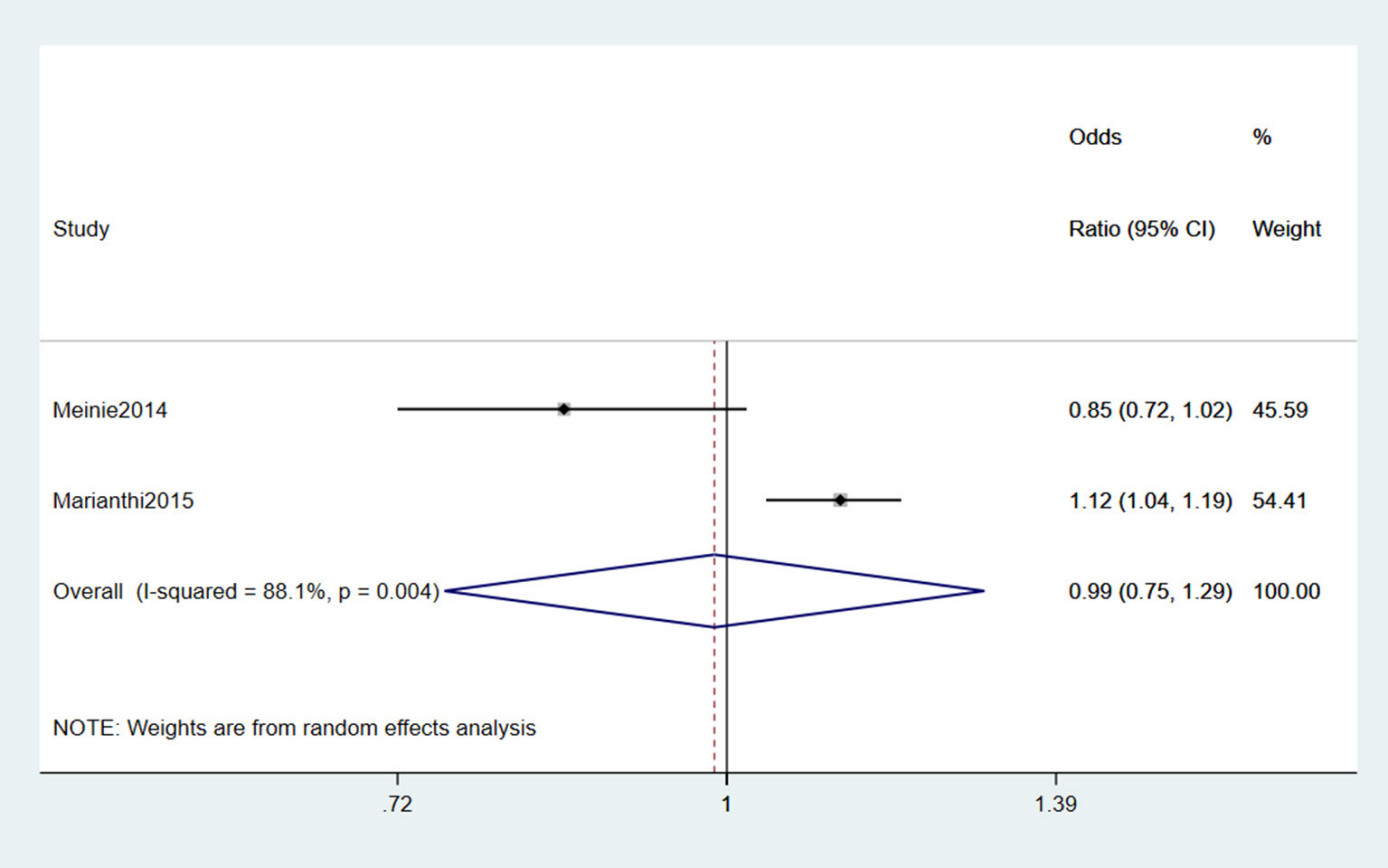
Forest plot of cerebrovascular disease.

Two studies (Seelen et al., 2014; Kioumourtzoglou et al., 2016) reported Cerebrovascular disease (CVD) as a risk factor for ALS. From the forest plot, an evident heterogeneity was detected (*I*^2^ = 88.1%), and the random effects model was performed for the meta-analysis. The results indicated that CVD was not a risk factor for ALS (OR = 0.99, 95% CI = 0.75, 1.29, *P* = 0.004; Figure 3). However, they did not classify the specific types of CVD. It is unknown which type of CVD has a greater impact on ALS.

#### 3.2.3. Diabetes mellitus

**Figure 4:**
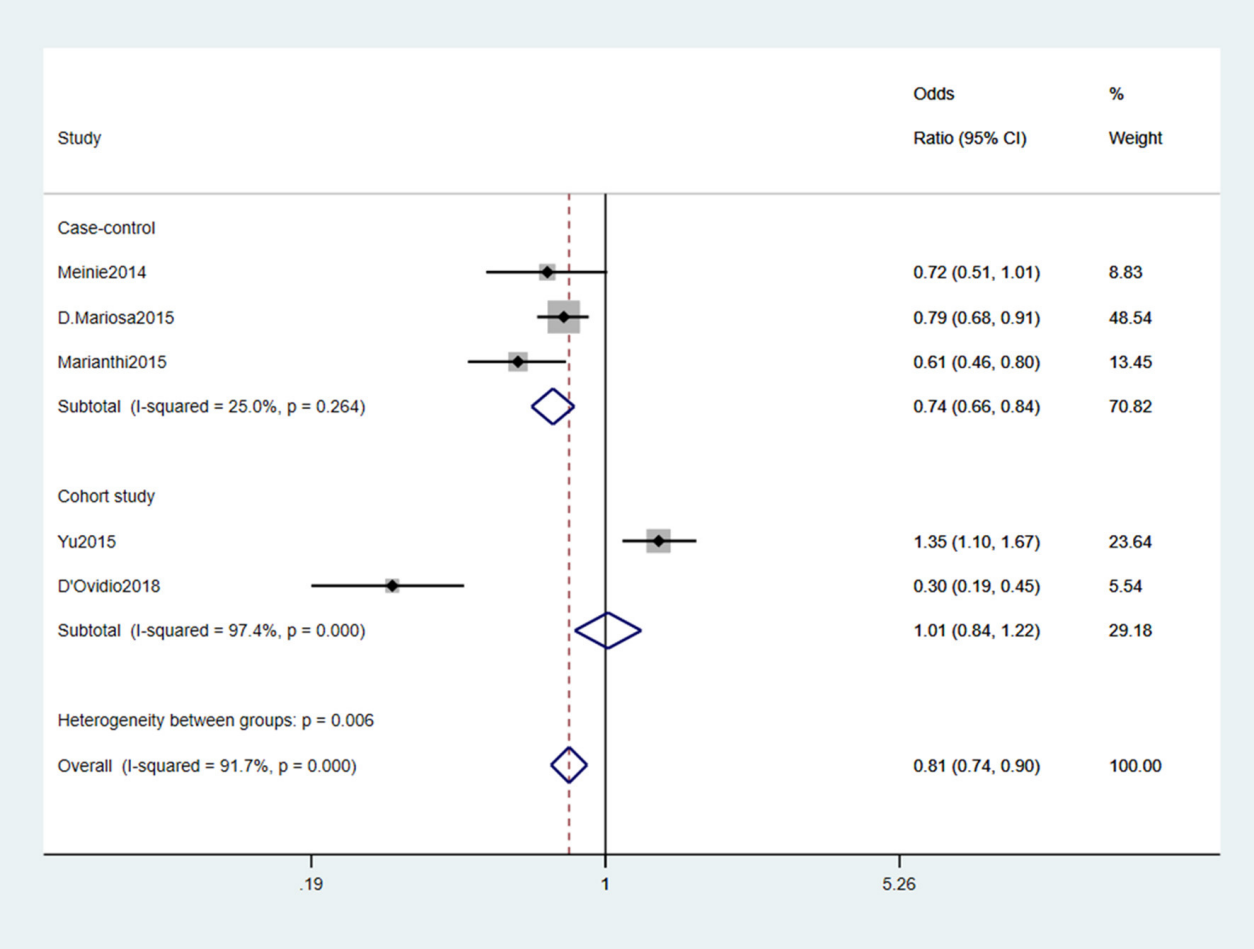
Forest plot of diabetes mellitus.

Five studies (Seelen et al., 2014; Kioumourtzoglou et al., 2015; Mariosa et al., 2015; Sun et al., 2015; D'Ovidio et al., 2018) reported DM as a risk factor for ALS. We analyzed the data and found significant heterogeneity (*I*^2^ = 91.7%). After reading the above five studies comprehensively, we found three case-control studies (Seelen et al., 2014; Kioumourtzoglou et al., 2015; Mariosa et al., 2015) and two cohort studies (Sun et al., 2015; D'Ovidio et al., 2018). Therefore, we performed the subgroup analysis by different study types. The results showed slight heterogeneity (*I*^2^ = 25%) in the case-control study group. A fixed-effects model was performed for meta-analysis. The results indicated that DM was a protective factor for the onset and progression of ALS (OR = 0.74, 95% CI = 0.66, 0.84, *P* = 0.264). Besides, the risk of developing ALS was 26% lower in those with DM than those without DM (Figure 4).

#### 3.2.4. Occupation

Among included studies, four (Das et al., 2012; Su et al., 2016; Peters et al., 2017; Filippini et al., 2020) reported the effect of occupation on the risk of developing ALS. Based on the characteristics of the occupation, we stratified the occupations into agriculture, industry, and services and performed statistical analyses.

(1) Agriculture

**Figure 5:**
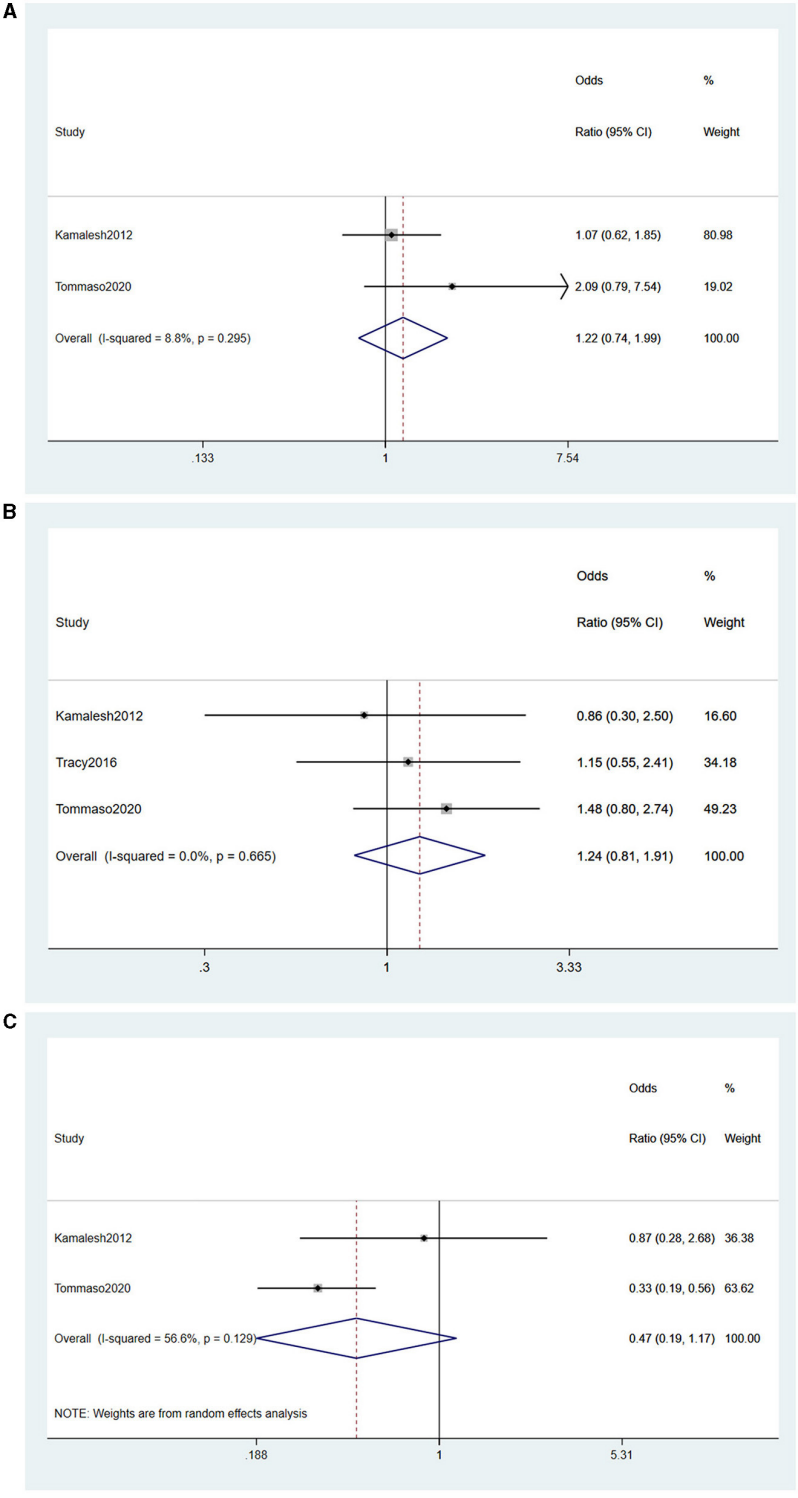
**(A)** Forest plot of occupation in agriculture. **(B)** Forest plot of occupation in industry. **(C)** Forest plot of occupation in service industry.

Two studies (Das et al., 2012; Filippini et al., 2020) reported agriculture as a risk factor for ALS, and no heterogeneity was detected (*I*^2^ = 8.8%). A fixed-effects model was performed for meta-analysis. Based on the forest plot, agriculture was not a risk factor for ALS (OR = 1.22, 95% CI = 0.74, 1.99, *P* = 0.295; Figure 5A).

(2) Industry

Three studies (Das et al., 2012; Peters et al., 2017; Filippini et al., 2020) reported industry as a risk factor for ALS. No heterogeneity was detected (*I*^2^ = 0%), and a fixed-effects model was performed for meta-analysis, concluding that industry was not a risk factor for ALS (OR = 1.24, 95% CI = 0.81, 1.91, *P* = 0.665) (Figure 5B).

(3) Service

Two studies (Das et al., 2012; Su et al., 2016) reported the service industry as a risk factor for ALS. Moderate heterogeneity was detected (*I*^2^ = 56.6%), and a random-effects model was performed for meta-analysis. From the forest plot, we could learn that the service industry was not a risk factor for ALS (OR = 0.47, 95% CI = 0.19, 1.17, *P* = 0.129; Figure 5C).

In summary, occupations including agriculture, industry, and services were not risk factors for ALS onset and progression.

#### 3.2.5. Smoking

**Figure 6:**
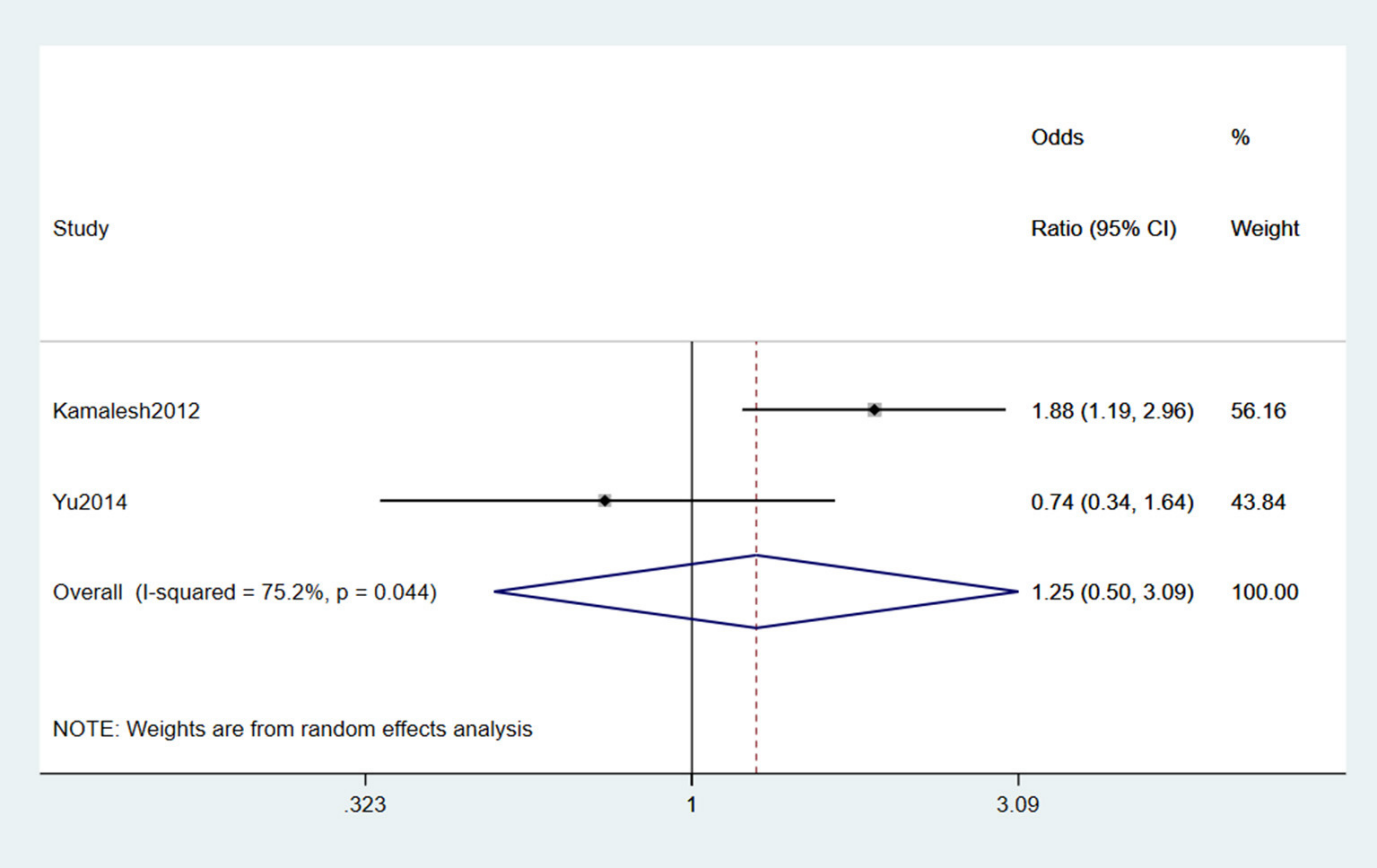
Forest plot of smoking.

Two studies (Das et al., 2012; Yu et al., 2014) reported smoking as a risk factor for ALS. Significant heterogeneity was detected (*I*^2^ = 75.2%), and a random-effects model was performed for the meta-analysis. The result indicated that smoking was not a risk factor for ALS (OR = 1.25, 95% CI = 0.5, 3.09, *P* = 0.044; Figure 6).

#### 3.2.6. Physical activity

**Figure 7:**
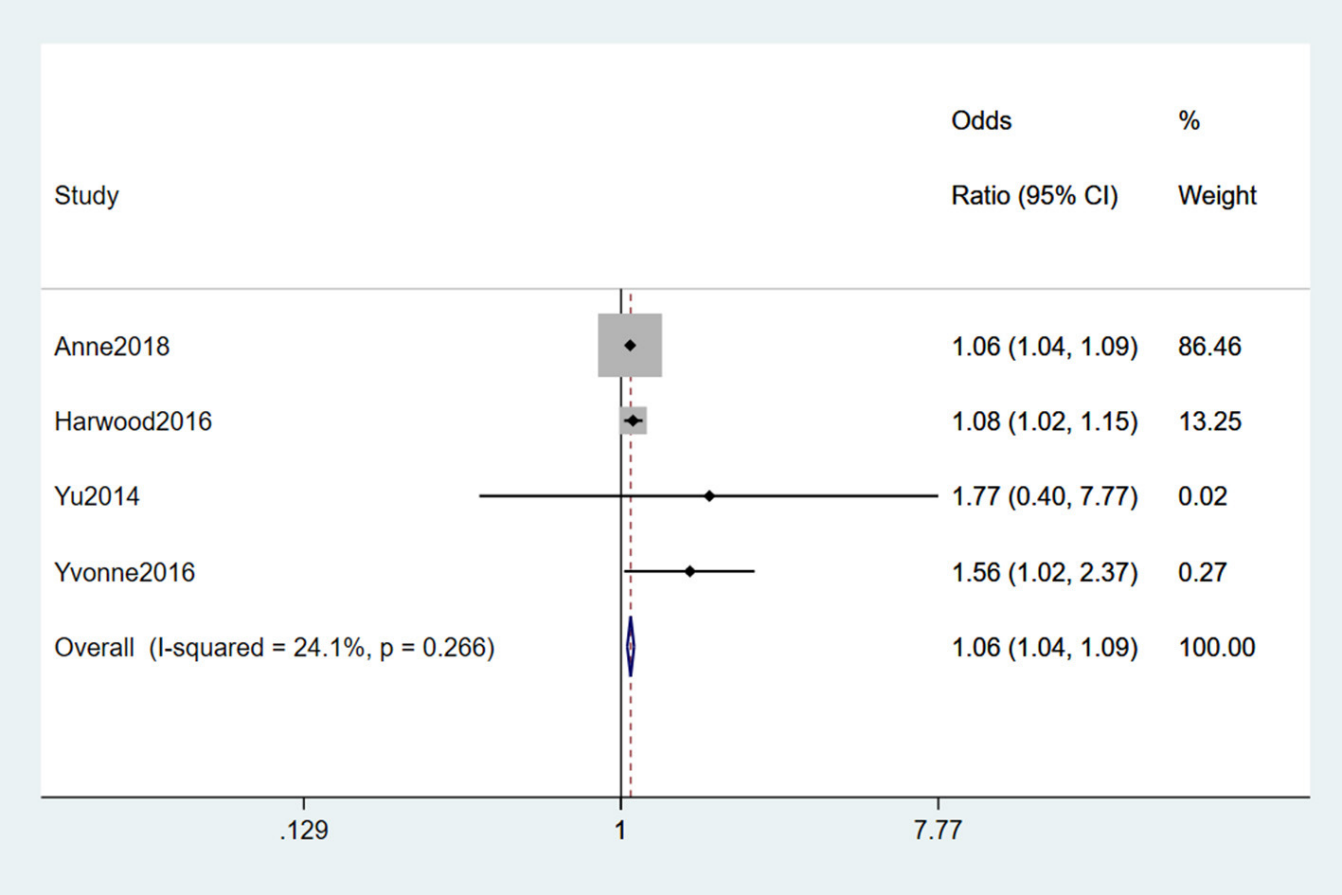
Forest plot of physical activity.

Six studies (Yu et al., 2014; Eaglehouse et al., 2016; Harwood et al., 2016; Visser et al., 2018; Rosenbohm et al., 2021) reported physical activity as a risk factor for ALS. Significant heterogeneity was detected (*I*^2^ = 91.7%). To find the source of heterogeneity, we performed sensitivity analysis and found that Rosenbohm et al. (2021) was the source, and the heterogeneity was significantly reduced after removing the study (*I*^2^ = 24.1%). Finally, a fixed-effects model was chosen for meta-analysis, and vigorous physical activity was considered a risk factor for ALS (OR = 1.06, 95% CI = 1.04, 1.09, *P* = 0.266). Moreover, we concluded that about 1.06 times higher as much as in those who did not take the vigor exercise (Figure 7).

#### 3.2.7. Electric shock

**Figure 8:**
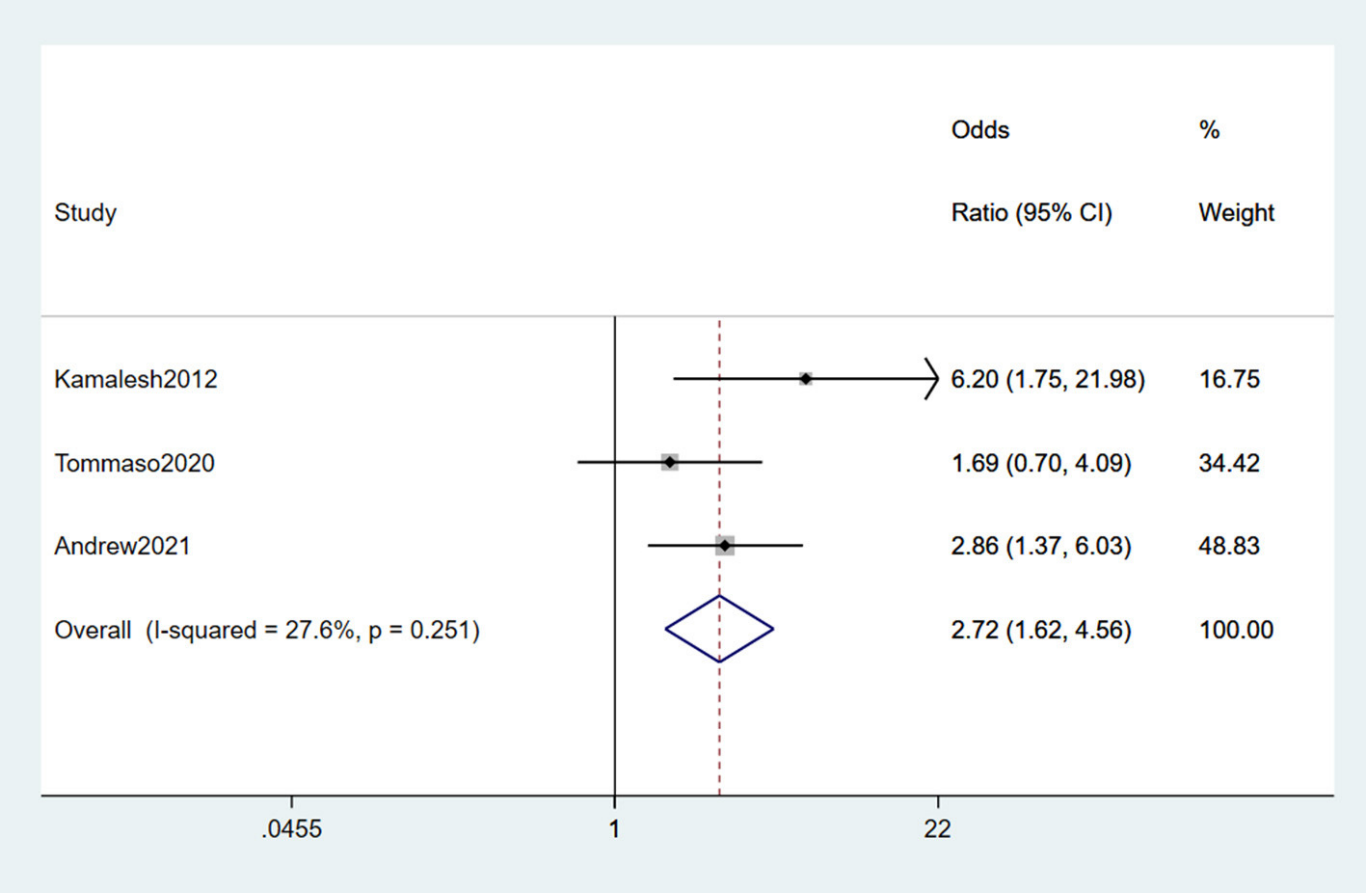
Forest plot of electric shock.

After comprehensively reading the included studies, electric shock is defined as people suffering from electric injury or living in a long-term electromagnetic field. Three studies (Das et al., 2012; Filippini et al., 2020; Andrew A. et al., 2021) reported electric shock as a risk factor for ALS, no heterogeneity was found among the findings (*I*^2^ = 27.6%). A fixed-effects model was chosen for the meta-analysis. From the result, we concluded that electric shock was a risk factor for ALS (OR = 2.72, 95% CI = 1.62, 4.56, *P* = 0.251), which indicated the risk of suffering from ALS in electric shock environments was 2.78 times higher than in non-electric shock environments (Figure 8).

#### 3.2.8. Military service

**Figure 9:**
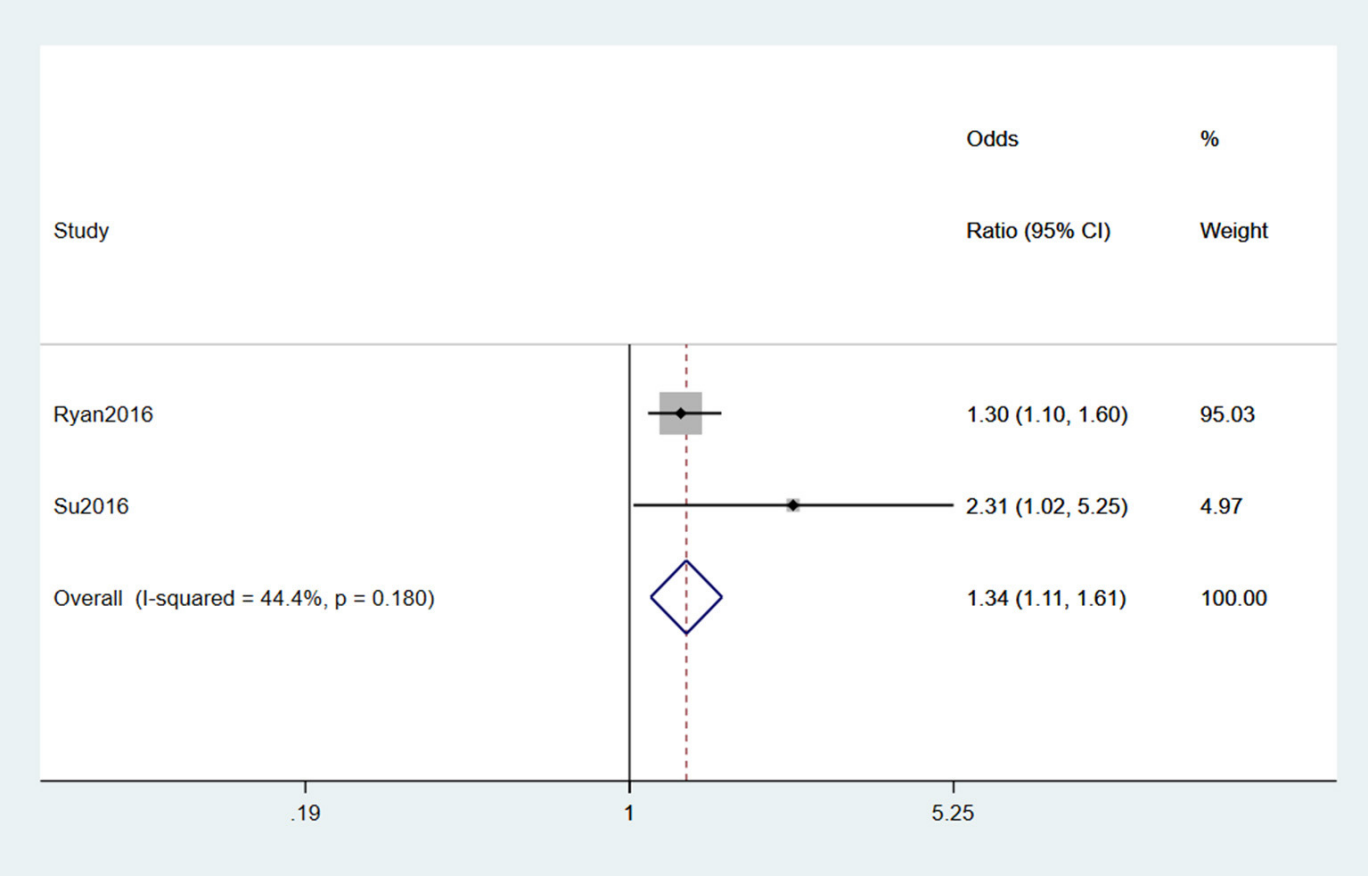
Forest plot of military service.

Two studies (Seals et al., 2016b; Su et al., 2016) reported military service as a risk factor for ALS. Moderate heterogeneity was detected (*I*^2^ = 44.4%), and a fixed-effects model was selected for meta-analysis. From the result of the forest plot, military service was a risk factor for ALS (OR = 1.34, 95% CI = 1.11, 1.61, *P* = 0.18), and those with military service had 1.34 times the risk of ALS of those who did not experience it (Figure 9).

#### 3.2.9. Pesticides

**Figure 10:**
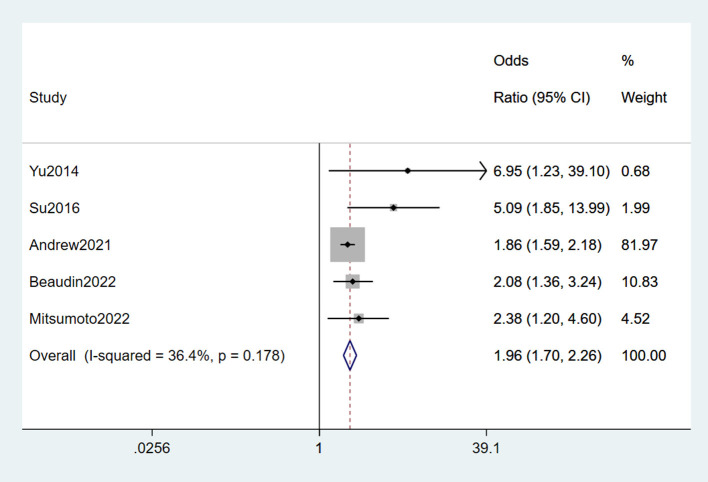
Forest plot of pesticides.

Five studies (Yu et al., 2014; Su et al., 2016; Andrew A. et al., 2021; Beaudin et al., 2022; Mitsumoto et al., 2022) reported pesticides as a risk factor for ALS. Slight heterogeneity was detected (*I*^2^ = 36.4%), and a fixed-effects model was selected for meta-analysis. From the forest plot, we concluded that pesticide was a risk factor for ALS (OR = 1.96, 95% CI = 1.7, 2.26, *P* = 0.178). In addition, the risk of suffering from ALS in those exposed to pesticides was 1.96 times higher than in those who were not (Figure 10).

#### 3.2.10. Lead

**Figure 11:**
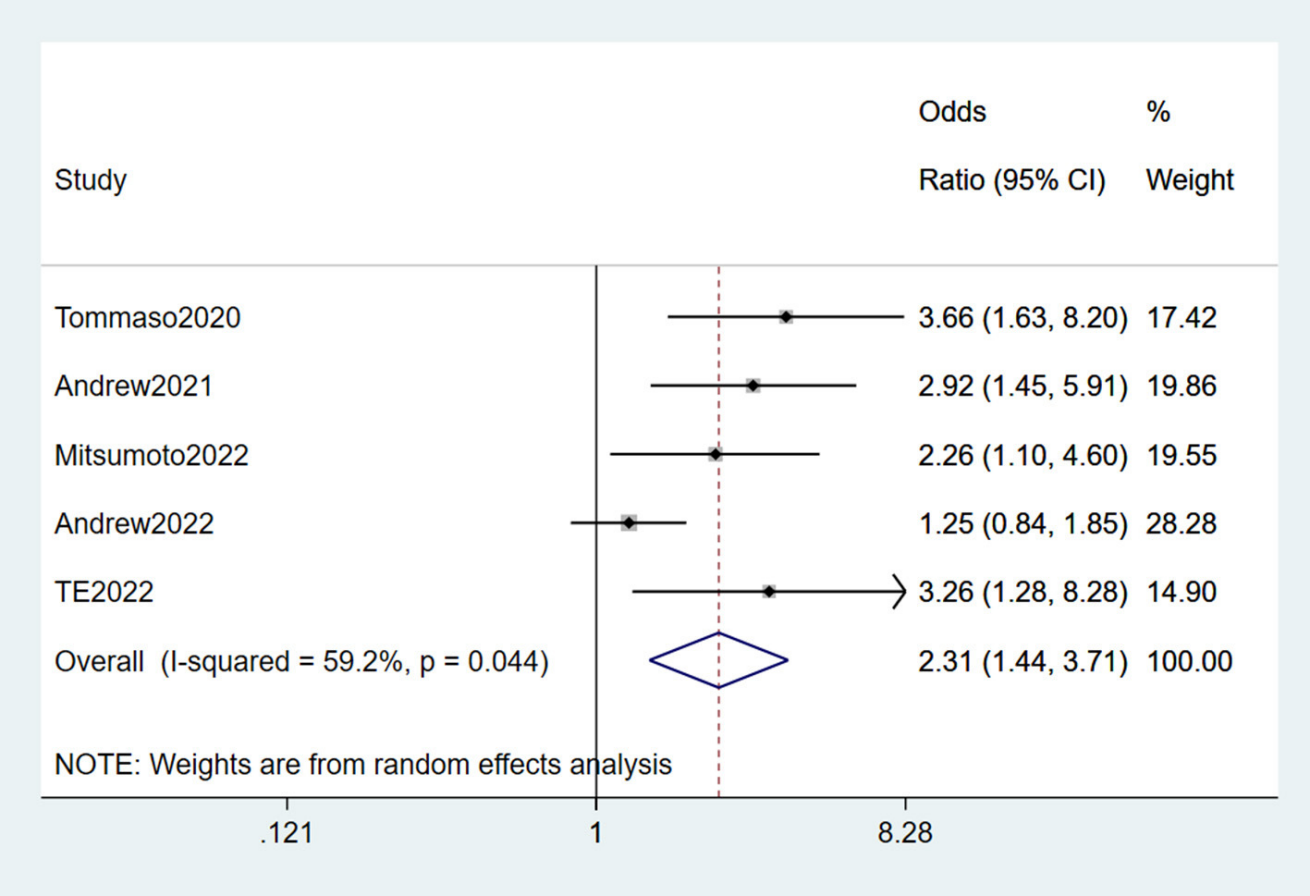
Forest plot of lead.

Six studies (Su et al., 2016; Filippini et al., 2020; Andrew A. S. et al., 2021; Andrew et al., 2022; Mitsumoto et al., 2022; Wang et al., 2023) reported lead as a risk factor for ALS. Significant heterogeneity was detected (*I*^2^ = 75.9%). Based on the sensitivity analysis result, Su et al. (2016) is the source of heterogeneity. After removing it, the heterogeneity was lower than before (*I*^2^ = 59.2%, *P* = 0.044 < 0.05), though it was still present. A random-effects model was performed for meta-analysis. We concluded that lead was a risk factor for ALS (OR = 2.31, 95% CI = 1.44, 3.71, *P* = 0.044). The risk of ALS in those exposed to lead was 2.31 times higher than in those not exposed (Figure 11).

#### 3.2.11. Chemicals

**Figure 12:**
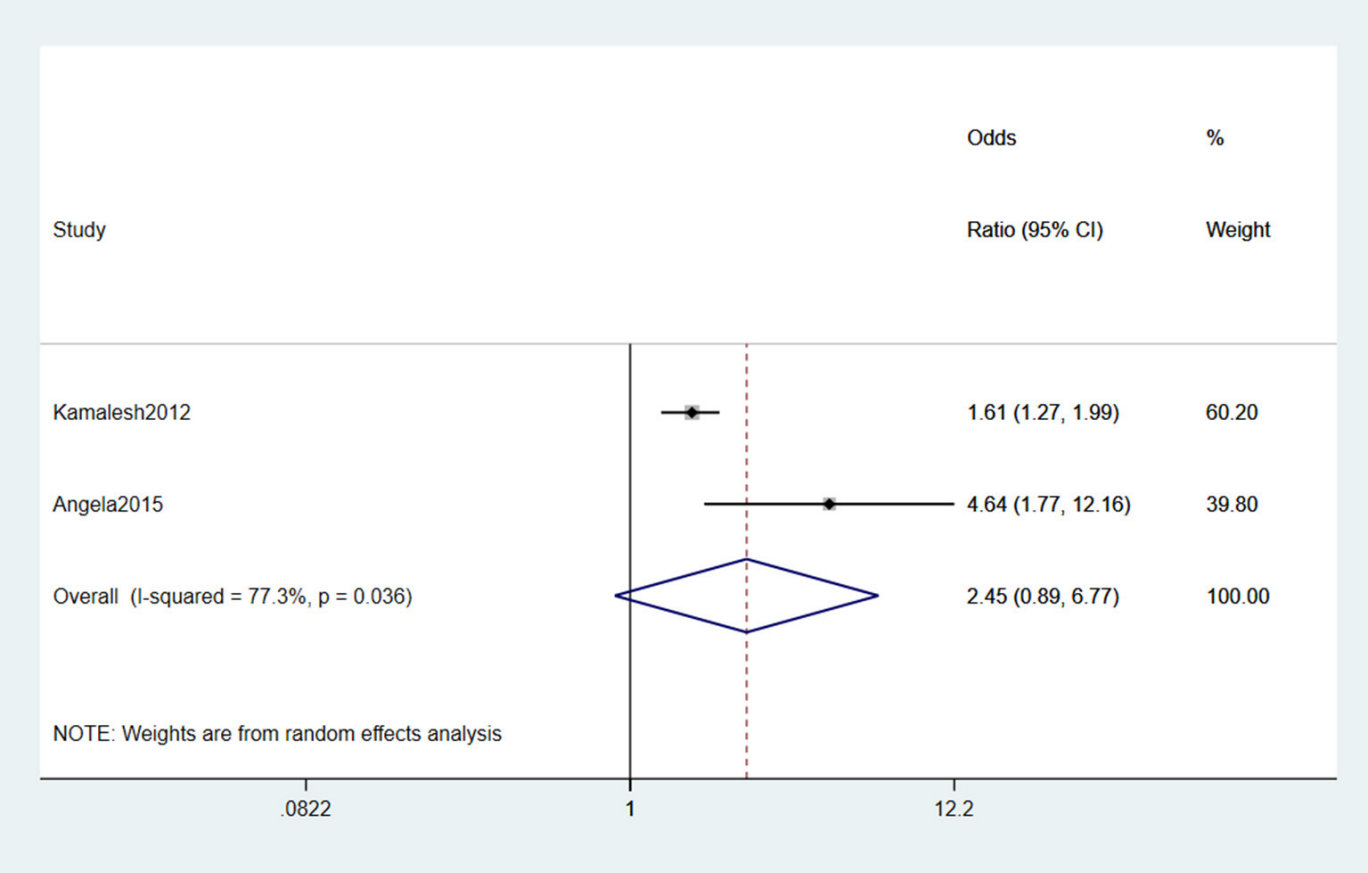
Forest plot of chemicals.

Two studies (Das et al., 2012; Malek et al., 2015) reported that chemicals were risk factors for ALS. Significant heterogeneity was detected (*I*^2^ = 77.3%) according to the forest plot. Moreover, the random effect model was selected for meta-analysis. In conclusion, the chemical agent was not a risk factor for ALS (OR = 2.45, 95% CI = 0.89, 6.77, *P* = 0.036; Figure 12).

#### 3.2.12. Heavy metal

**Figure 13:**
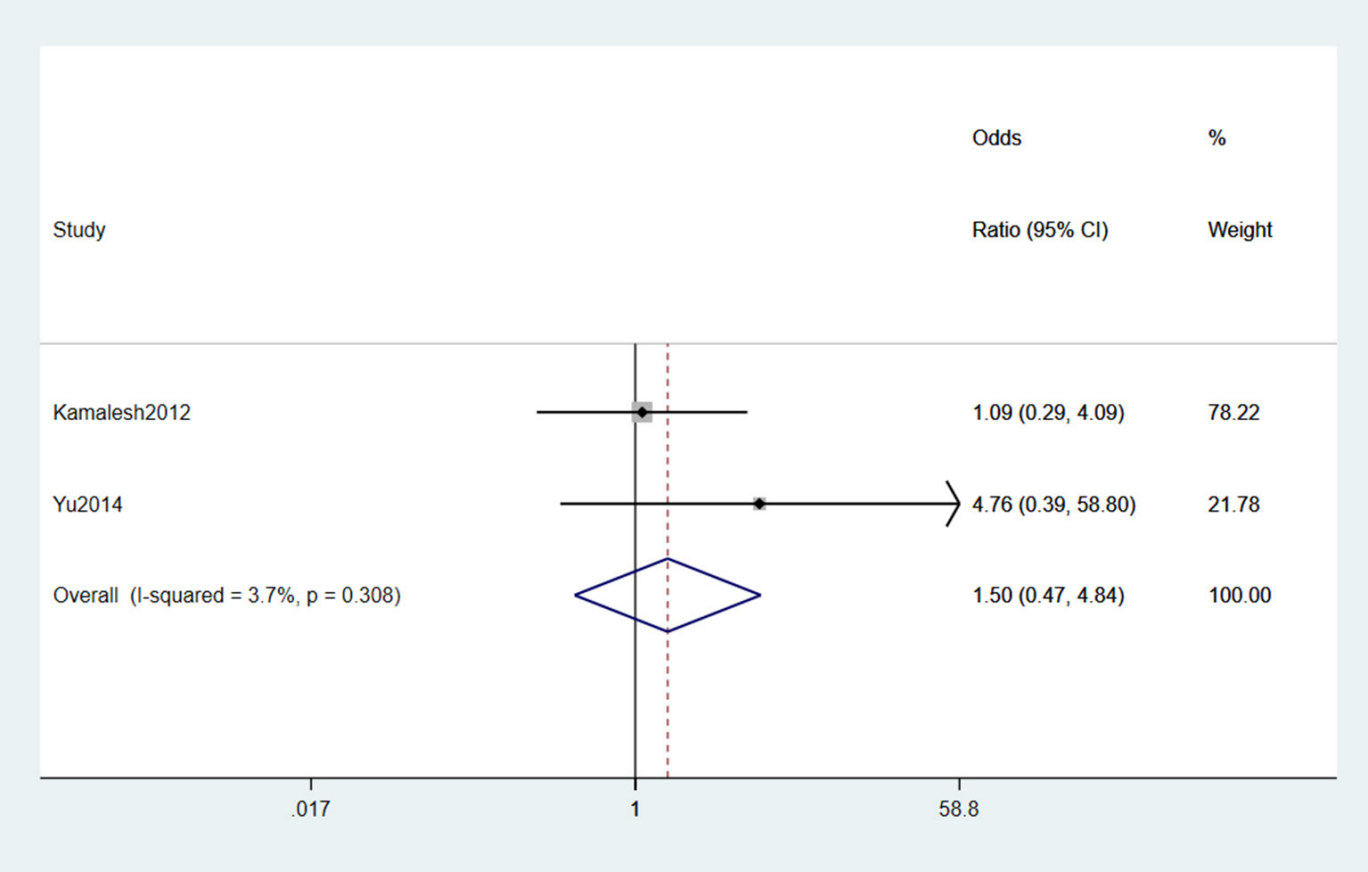
Forest plot of heavy metal.

In two studies (Das et al., 2012; Yu et al., 2014) reported that heavy metals were risk factors for ALS. However, the original studies did not report the definition and classification of heavy metals. No heterogeneity was detected according to the forest plot (*I*^2^ = 3.7%), and the fixed-effects model was selected for meta-analysis. Based on the statistical result, we concluded that heavy metals were not risk factors for ALS (OR = 1.5, 95% CI = 0.47, 4.84, *P* = 0.308; Figure 13).

#### 3.2.13. Other risk factors

The remaining 17 risk factors were hypotensor, educational status, residential, air pollution, steroid, service industry, high metabolism, water resources, statin, BMI, alopecia, aspirin, trace metals in blood, intestinal biopsy, nutrition, lipid metabolism, heavy metal, and FVC. However, these risk factors were only reported in one independent study and thus not be analyzed. The details of these risk factors are shown in Table 2.

## 4. Discussion

Thirty-six observational studies were included in this study, 11 of which were cohort studies, and the rest were case-control studies. In this meta-analysis, we included high-quality observational studies by NOS quality assessment to ensure the quality of evidence. The scores of the included studies were more than seven grades. With the low incidence of ALS defined as a rare disease and the unclear mechanism, randomized controlled trials (RCT) were not feasible. Observational studies, as a component of epidemiological studies, are effective study types for exploring risk factors for the disease. This study aimed to explore the risk factors for ALS and perform a systematic review and meta-analysis based on observational studies.

We used STATA 15.0 software and Review Manager 5.3 for meta-analysis. Based on the statistical analyses, we concluded that head trauma, physical activity, electrical shock, military service, pesticides, and lead were related to the onset and progression of ALS, but diabetes mellitus was its protective factor.

In this study, head trauma was a risk factor for ALS, and the relationship between the progression of ALS and head trauma has drawn attention among researchers. Head trauma is known to result in neurodegenerative diseases and cognitive impairment (Gupta and Sen, 2016). In a previous meta-analysis, head injury was identified as a risk factor for the development of ALS (Chen et al., 2007). However, after adjusting the time difference between the time of head trauma and the time of ALS onset, no direct association between the two groups. Schmidt et al. (2010) conducted a cohort study among veterans and found that patients with a history of head trauma within 15 years before the record date had a higher risk of developing ALS (OR= 2.33, 95% CI = 1.18–4.61). However, after reading the included studies, we found that one study (Andrew A. et al., 2021) reported the frequency of head trauma associated with the onset of ALS. The study analyzed one and multiple injuries of head trauma frequency. The aim was to explore in-depth the relationship between head trauma and ALS. Further dose-response and stratification analyses are needed to determine whether head trauma is associated with the site, number, and severity of the injury, and more large-sample, prospective cohort studies are needed for evidence support.

According to the result of meta-analysis, CVD is not a risk factor for ALS. CVD consists of various diseases of the brain blood vessels, which present hemorrhagic or ischemic vessels in the brain tissue. Researchers have now found that alterations in vascular homeostasis may be relevant to the pathogenesis of ALS (Sutedja et al., 2011). Low levels of vascular endothelial growth factor are more likely to lead to the degeneration of motor neurons (Goncalves et al., 2017). This study revealed that total CVD is not a risk factor for the progression of ALS based on the statistical analysis. However, Kioumourtzoglou et al. (2016) found a protective effect of atherosclerosis and ischemic heart disease on the progression of ALS.

DM is a protective factor for ALS in statistical analysis. The current mechanism of DM in ALS focuses on the following hypotheses. DM can resist the increased energy expenditure and high metabolism in ALS patients and play a protective role, which delays the onset of ALS (Blasco et al., 2017). Secondly, DM is associated with high concentrations of granule protein precursors, a biomarker associated with TDP-43-mediated axonopathy, and the abnormal protein of TDP-43 aggregation is one of the significant risk factors for ALS (Cieslarova et al., 2017). Therefore, it can be hypothesized that DM may act through TDP-43. In this meta-analysis, by subgrouping different study types, the results of the case-control studies showed a moderate protective effect of DM on the onset and progression of ALS. After comprehensively reading the full texts of the five included studies, we found that only one study (Seelen et al., 2014) performed a correlation analysis between autoimmune diseases and ALS, but the results showed no statistical significance. However, better protection was not demonstrated in the cohort studies. In this study, the result of cohort and case-control studies were inconsistent. A US cohort study found that patients with DM before onset had a later onset, while having T2DM in an older age group may reduce the risk of developing ALS, while having T1DM in a younger age group may increase the risk of developing ALS (Hollinger et al., 2016). Thus, this may be a source of study heterogeneity. Therefore, more prospective cohort studies with larger samples are needed to demonstrate this.

The occupation was not a risk factor for ALS. The included studies were divided into agriculture, industry, and services industry based on the nature of the occupation because of its evident heterogeneity. It was finally concluded that none of the three significant occupations was a risk factor for the onset and progression of ALS. It has been pointed out that people engaged in agriculture and industry are more likely to be exposed to pesticides, heavy metals, tobacco, and other neurotoxic substances, and the combined effect of these factors may be the main reason for the increased risk of the disease in this group (Saastamoinen et al., 2022). Therefore, we further explored whether pesticides, lead, chemicals, and heavy metals were risk factors for ALS. With statistical analyses, we found that pesticides and lead were risk factors for the progression of ALS, while heavy metals and chemicals were not among their risk factors. Lead has potent neurotoxicity, mainly in the central nervous system (CNS) and peripheral nervous system (PNS), with CNS toxicity being the most obvious. Some studies have found that short-time exposure to high lead concentrations may cause subacute changes in motor neurons (Thomson and Parry, 2006). The pathogenic mechanisms of pesticide-induced neurological damage are well-understood. Cholinesterase inhibition, polymorphism of paraoxonase, and induction of oxidative stress are the leading causes of neurological damage caused by pesticides. In this study, we did not find an evident relationship between heavy metals and chemicals and the onset of ALS, while many studies have concluded that heavy metal exposure is not associated with the risk of developing ALS (Vinceti et al., 2012; Nicoletti et al., 2016). However, this study did not analyze the heavy metal type and exposure duration as covariates. Further studies are needed to determine whether there is a correlation between the covariates and ALS.

Smoking was not a risk factor for ALS. Smoking history was regarded as the primary differentiation method, which may ignore the effects of confounders such as dose and gender differences. Neurotoxic components such as nicotine and formaldehyde produced during tobacco combustion are the leading cause of the increased risk of ALS development, inhibiting oxy phosphatase activity and leading to oxidative stress of cellular DNA, leading to apoptosis (Thorne et al., 2009). Furthermore, we explored the reason for lacking a significant result, and we concluded that one of the included studies had a small sample size, which was not representative, and it did not adjust for confounders.

Physical activity was a risk factor for ALS. Physical activity causes the body to produce more reactive oxygen species, leading to nucleotide damage, increasing the oxidative load on cells, and cellular damage. A Mendelian randomization study found a causal relationship between the heritability of frequent and vigorous exercise and ALS (Julian et al., 2021).

Electric shock was a risk factor for ALS. The nervous system is one of the sensitive systems of the body in response to external environmental stimuli and a target for exposure to environmental electromagnetic fields. Several studies have found a correlation between occupational exposure to low-frequency magnetic fields and the risk of developing neurodegenerative diseases (Rinaldi et al., 2015; Kopeikina and Ponomarev, 2021). In this study, data analysis revealed that people with long-term EMF exposure were 2.72 times more likely to develop ALS than non-exposed people.

Military service was a risk factor for ALS. Multiple exposures may occur during military service, including pesticides, head trauma, viral infections, organic solvents, and formaldehyde (McKay et al., 2021). These exposures may be suggested to be a linkage to an increased risk of ALS. This study only examines whether there is an association between ALS and military service, and further analyses of possible exposure factors in military service should be conducted in order to be able to improve the level of evidence. Of note, both included studies describe military service as a type of occupation. In order to standardize the occupational factors, we divided the occupations into agriculture, industry, and services according to their nature. For more detailed findings, we conducted separate meta-analyses of the two studies on military service.

Chemicals were not a risk factor for ALS. A review of the full text of the included literature revealed that the chemical agents in this study were neurotoxic substances, including aromatic and organochlorinated solvents. Chronic exposure to chemical agents can cause heterogeneous metabolic pathways, and one study found that (Weisskopf et al., 2009) the genotype of glutathione synthase interacts with chemicals such as solvents to increase the risk of developing ALS.

The remaining 17 factors, which were only reported once and could not be performed in meta-analyses, may be a risk or protective factor for the development and progression of ALS. We do hope that more high-quality observational studies will be included in the meta-analysis in the future to provide strong evidence to support the results.

The strengths of this study lie in the comprehensive analysis of all risk factors for ALS in recent years and the inclusion of high-quality observational studies by NOS quality scores to ensure the credibility of the evidence. Secondly, we included retrospective case-control and prospective cohort studies to provide a comprehensive view of the relationship between risk factors and ALS. Third, we performed subgroup and sensitivity analyses to provide more reliable estimates. Finally, our study included participants of various nationalities, considering genetic differences due to ethnicity. A collation of the included studies revealed that 17 were targeted at European populations, 14 at North American populations, and five at Asian populations. However, our study also has some limitations. First, we included observational studies, which are not scientifically designated compared to the randomized controlled trials (RCTs) category. Of note, due to the limited number of studies, five risk factors, including CVD, smoking, military, chemical, and heavy metal, only had two studies when meta-analyses were performed. Nevertheless, the PRISMA guideline recommends that a meta-analysis of one outcome be conducted with more than three studies. Under this circumstance, we should interpret the results with caution. We hope more relevant and high-quality observational studies will be included and provide a more reliable result. In addition, although ORs with fully adjusted models were used in our study, confounders varied across studies and may have had some degree of influence on the results. Finally, the only included studies were papers that could be published in the public domain, so we need to consider the effect of publication bias on the results of this study.

Besides, we classified the risk factors into lifestyle, underlying disease history, and occupation according to the nature of the risk factors. Physical activity, electric shock, and smoking could be defined as a lifestyle. CVD, DM, and head trauma are defined as underlying diseases. Lead, pesticides, military service, chemicals, and heavy metals are considered occupations. Due to inconsistencies between studies, effect sizes could hardly be combined according to the inclusion and exclusion criteria. However, we still hope that interaction and possible superpositions effects between a class of factors could be analyzed with more high-quality studies.

## 5. Conclusion

Taken together, we investigated the following risk factors associated with the onset and progression of ALS: head trauma, physical activity, electric shock, military service, pesticides, and lead. However, in meta-analyses, CVD, occupation, smoking, chemicals, and heavy metals were not associated with ALS onset and progression. In addition, DM was considered as a protective factor, which helped patients with DM complicated by ALS to assess their prognosis better. Moreover, we look forward that more longitudinal studies will be conducted to further elucidate the relationship between the type of DM and ALS onset and progression.

## Data availability statement

The original contributions presented in the study are included in the article/Supplementary material, further inquiries can be directed to the corresponding authors.

## Author contributions

BC and DX contributed to the studies retrieval and data extraction. QZ and YZ designed and developed the framework for the manuscript. JZ and YZ analyzed the data. HH and JH manipulated the software. All authors contributed to the article's writing, read the manuscript, and agreed to submit this version.
