# Supplementary material for: Risk factors associated with amyotrophic lateral sclerosis based on the observational study: a systematic review and meta-analysis
Source: Front Neurosci. 2023 May 22;17:1196722. doi: 10.3389/fnins.2023.1196722 (PMC10239956; doi:10.3389/fnins.2023.1196722)
Supplement: Supplementary file 1 [file Table_1.docx]

| Study | Selection | Comparability | Exposure | Total |
| --- | --- | --- | --- | --- |
| Kamalesh2012 | ⭐⭐⭐ | ⭐⭐ | ⭐⭐⭐ | 8 |
| TRACY2012 | ⭐⭐⭐⭐ | ⭐⭐ | ⭐⭐⭐ | 9 |
| Elinor2013 | ⭐⭐⭐ | ⭐⭐ | ⭐⭐ | 7 |
| Yu2014 | ⭐⭐⭐ | ⭐ | ⭐⭐⭐ | 7 |
| Meinie2014 | ⭐⭐⭐ | ⭐⭐ | ⭐⭐ | 7 |
| Feng2015 | ⭐⭐⭐⭐ | ⭐⭐ | ⭐⭐⭐ | 9 |
| Ching2015 | ⭐⭐⭐⭐ | ⭐⭐ | ⭐⭐⭐ | 9 |
| D.Mariosa2015 | ⭐⭐⭐ | ⭐⭐ | ⭐⭐⭐ | 8 |
| Marianthi2015 | ⭐⭐⭐⭐ | ⭐⭐ | ⭐⭐⭐ | 9 |
| Marianthi2015 | ⭐⭐⭐⭐ | ⭐⭐ | ⭐⭐⭐ | 9 |
| Mark2015 | ⭐⭐⭐⭐ | ⭐⭐ | ⭐⭐⭐ | 9 |
| Angela2015 | ⭐⭐ | ⭐⭐ | ⭐⭐⭐ | 7 |
| Yu2015 | ⭐⭐ | ⭐⭐ | ⭐⭐⭐ | 7 |
| Ryan2016 | ⭐⭐⭐⭐ | ⭐⭐ | ⭐⭐⭐ | 9 |
| Su2016 | ⭐⭐⭐⭐ | ⭐⭐ | ⭐⭐⭐ | 9 |
| Peters2016 | ⭐⭐⭐⭐ | ⭐⭐ | ⭐⭐⭐ | 9 |
| Harwood2016 | ⭐⭐⭐⭐ | ⭐⭐ | ⭐⭐⭐ | 9 |
| Tracy2016 | ⭐⭐⭐⭐ | ⭐⭐ | ⭐⭐⭐ | 9 |
| Yvonne2016 | ⭐⭐⭐⭐ | ⭐⭐ | ⭐⭐⭐ | 9 |
| Ryan2016 | ⭐⭐ | ⭐⭐ | ⭐⭐⭐ | 7 |
| Anne2018 | ⭐⭐⭐ | ⭐⭐ | ⭐⭐ | 7 |
| D'Ovidio2018 | ⭐⭐⭐⭐ | ⭐⭐ | ⭐⭐ | 8 |
| Ola2019 | ⭐⭐⭐ | ⭐ | ⭐⭐⭐ | 7 |
| Tommaso2020 | ⭐⭐⭐⭐ | ⭐⭐ | ⭐⭐⭐ | 9 |
| Andrew2021 | ⭐⭐⭐⭐ | ⭐⭐ | ⭐⭐⭐ | 9 |
| Andrew2021 | ⭐⭐⭐⭐ | ⭐⭐ | ⭐⭐⭐ | 9 |
| Yu2021 | ⭐⭐⭐⭐ | ⭐⭐ | ⭐⭐ | 8 |
| Rosenbohm2021 | ⭐⭐⭐⭐ | ⭐ | ⭐⭐⭐ | 8 |
| Skajaa2021 | ⭐⭐⭐⭐ | ⭐⭐ | ⭐⭐⭐ | 9 |
| Thompson2021 | ⭐⭐⭐ | ⭐⭐ | ⭐⭐ | 7 |
| Sun2021 | ⭐⭐⭐ | ⭐⭐ | ⭐⭐⭐ | 8 |
| Beaudin2022 | ⭐⭐⭐⭐ | ⭐⭐ | ⭐⭐⭐ | 9 |
| Mitsumoto2022 | ⭐⭐⭐ | ⭐⭐ | ⭐⭐ | 7 |
| Andrew2022 | ⭐⭐⭐⭐ | ⭐⭐ | ⭐⭐⭐ | 9 |
| He2022 | ⭐⭐⭐⭐ | ⭐⭐ | ⭐⭐⭐ | 9 |
| TE2022 | ⭐⭐⭐⭐ | ⭐⭐ | ⭐⭐⭐ | 9 |

Supplementary table 1. NOS quality assessment
